# On the Introduction of Specialities into Bellevue Hospital

**Published:** 1863-05

**Authors:** Louis Elsberg

**Affiliations:** F.A.M.; Lecturer on the Diseases of the Larynx and Throat at the University


ON THE INTRODUCTION OF SPECIALTIES INTO BELLEVUE HOSPITAL.—The 20th of April, 1863, may prove to have been an important day in the medical history of our Public Institution. On that day the Commissioners of Charities and Correction, desiring to do their duty in advancing all proper means to place the poor who are obliged to seek refuge in the hospitals under their charge upon the best footing consistent with their condition, and make available all the developments under the advance and progress of medical science, took into consideration the subject of enlarging the facilities of Bellevue Hospital for the benefit of the poor of this city, for the treatment of specialties, and "in order to elicit all information in respect thereto," they addressed a letter of inquiry to the Medical Board. What answer will be given?

There was a time when all science, physical and metaphysical, could be grasped by one master-mind. While human knowledge was yet limited to but a small number of principles and facts, a few elect, like a Hippocrates or an Aristotle, could sway at the same time the sceptres of Medicine and Natural Science. But as knowledge more and more accumulated, division and subdivision became necessary. Thus medicine separated *as a specialty* from its fountain-head and co-ordinate streams, the physical sciences. Thus physicians were impelled to devote themselves to special departments of medical study or practice exclusively, until to-day each branching path of the vast domain is found to lead to a mine of inexhaustible research, in which one human lifetime is but a short working hour of the delver, and it is no longer permitted even to a Humbolt or a Virchow to embrace all the immense details of our science and art.* To preserve the natural unity of the subject, to accommodate the general wants of variously constituted communities, the bulk of the profession must perhaps ever remain "general practitioners," but no one can hope to obtain pre-eminence in any one branch without bestowing upon that branch special attention; and even the most arduous and toilsome worker must be content to contribute a few building blocks, or it may be but a single stonelet, to the grand temple for the healing of the afflicted which holds the common altar of all physicians, specialists and general practitioners. Specialism is not without dangers. The specialist must preliminarily be a regu-

* *Niemrnd ist im Stande, das ungeheure Detail unserer Wissenschaft gans zu ube hen.*"—VIRCHOW.

larly educated man; other things being equal, the best general practitioner, of course, becomes the best specialist. Division on the one hand may be carried into absurd extremes, and on the other not correspond to the actual advancement of our art. But the advantages of specialism to the patient and to science far overbalance its real and presumed injurious effects. It is well known, too, that the profession of the country has long looked with suspicion and disfavor upon the name "specialty," and the character of a "specialist." The origin of the prejudice is also well known; nor was it unreasonable or unjustifiable to associate the idea of quacksalver and knave with that of specialist, as long as no regularly educated honorable member of the profession took the title; as long as ignorant and unprincipled imposters monopolized the reputable name with a disreputable game at the expense of the health and pockets of the credulous portion of the community. But, happily, that time is past. Though pretenders abound to usurp all titles, that of specialist is proudly borne by many honorable men, men of genius and enlarged medical knowledge. Verily the day of professional progressive specialty is coming—nay, has already come. The step taken by the Commissioners of Charities and Correction is therefore a liberal and important one. May the improvement it inaugurates soon be realized for the benefit of the sick, and the advancement of science. The medical staff of Bellevue Hospital, and indeed the whole profession, will doubtless heartily co-operate in the praiseworthy endeavors of the Commissioners; and whatever be the precise expression of individual views and opinions in the answer of the Board, its spirit and general import may, I think, safely be predicted!

But the Commissioners, after mentioning eye, ear, nervous and skin diseases, request a reply also to the following intergatory:

2. Are there other diseases *and Specialties* that should be regarded as important to *treat by themselves* under similar provision?

THROAT DISEASES, *especially affections of the larynx*, should in answer be prominently brought forward to the Board. The frequency of their occurrence; their leading to the most serious consequences in many, and to considerable inconvenience in all cases, either terminating fatally, and often with much rapidity, or intailing difficulties of the voice, the respiration, and deglutition; the fact that the use of the laryngoscope for their recognition and treatment has recently revolutionized our knowledge of them, having transferred them from the category of *internal* and obscure to that of *external* diseases within our reach, and the little attention given thereto as yet by the profession at large; render them peculiarly suitable for such separate treatment. There is perhaps no class of patients to which even the most accomplished physicians do less justice than to the sufferers from laryngeal affections. Of this there is ample evidence afforded me at the *Clinic*, which the liberality of the Faculty of the University Medical College has enabled me to establish, and, as far as I have been informed, the experience of Bellevue Hospital proves the same thing.

I conclude with the words of the illustrious Berlin and Utrecht Professsors (Virchow and Donders), "To specialists must we look for further advancement of the healing art!" —"Specialties nowadays are the essential conditions of the progress in science. * * * * Whoever speaks of progress in medicine to-day must needs speak of specialties!"

LOUIS ELSBERG, M.D., F.A.M.,
*Lecturer on the Diseases of the Larynx and Throat at the University.*
156 West 15th street.

[*American Medical Times.*

